# A Rarer Case of Spontaneous Coronary Artery Dissection causing Acute Coronary Syndrome in a Puerperal Woman: Case Report

**DOI:** 10.51894/001c.6435

**Published:** 2017-12-19

**Authors:** Milad Asfar

**Affiliations:** 1 McLaren Macomb, PGY2, Emergency Medicine

**Keywords:** coronary artery dissection, acute coronary syndrome, catheterization, myocardial infarction

## Abstract

Chest pain is one of the most common complaints of patients presenting to the emergency room department. There are many causes of chest pain and providers must always consider acute coronary syndrome (ACS). Spontaneous coronary artery dissection is a very rare cause of ACS but providers must consider this possibility in younger patients without risk factors or a history of coronary artery disease. This rare phenomenon is commonly associated with pregnant women or those in the postpartum period. This is a case report study describing a classic scenario of a postpartum female in her early 30s who presented with spontaneous coronary artery dissection. Providers obtained the usual tests of an electrocardiogram (EKG) and blood work. She then had a heart catheterization performed. The patient was conservatively managed through use of medications without any use of invasive measures. The patient also had other images of the chest taken to rule out other causes of chest pain such as pneumothorax and pulmonary embolism. There were EKG changes noted, as well elevated troponin levels suggestive of heart damage. The heart catheterization results showed coronary artery dissection involving the artery that correlated with the EKG changes. Spontaneous coronary artery dissection is a rare phenomenon that can present as ACS. Pregnant and postpartum women are at higher risk to develop this condition. It is essential to consider this condition along with other causes of chest pain.

## INTRODUCTION

Spontaneous coronary artery dissection is defined as a non-traumatic and non-iatrogenic condition resulting in dissection of the coronary arterial wall.^1^ The mechanism of this rare disease is not well known, but there are a few suggestions that have been proposed previously. A tear in the intimal layer may lead to creation of true and false arterial lumens. The artery is made of three layers known as the intima, media and adventitia. The inner layer is the intimal layer that may develop a tear leading to the dissection. Another thought is bleeding of the vasa vasorum (i.e., the blood vessels that supply the media and adventitia layers) while the intima remains intact resulting in intramural hematoma.^2^

The estimated occurrence of acute coronary syndrome (ACS) from spontaneous coronary artery dissection in the general population is 0.1 to 0.4 percent.^3^ This percentage is much larger when considering the condition as a cause of ACS in women especially those whom are young with ages below 50 at or around the peripartum period. In a recent study, spontaneous coronary artery dissection was found to be responsible for 22.5% of women younger than 60 years old with ACS findings. The patient in this case report demonstrates a classic presentation of a patient who would have a spontaneous coronary artery dissection.

### Case Report

The patient in this case report is a female in her early 30s who presented to the emergency department at McLaren Macomb medical center for evaluation of chest pain. The pain started suddenly an hour and a half prior to her arrival to the hospital while she was sitting on the couch. She admitted to two episodes of vomiting as well as bilateral upper extremity paresthesias but denied any other symptoms. Her paresthesias symptoms were numbness and tingling that occurred on and off since her onset of chest pain. She has a recent history of childbirth by vaginal delivery approximately two weeks prior to presentation. Her pregnancy and delivery were uneventful and she had been doing well prior to the symptoms that started on this day.

The patient had a past medical history that consisted of anemia and hypothyroidism. She had a previous surgery history of dilation and curettage but denied other surgeries. Her only home medication was a thyroid medication. She denied any history of tobacco, alcohol, illicit drug use or any history of risk factors for cardiac disease. Physical examination of the patient did not show any significant findings. Her vitals were within normal limits and she was in no acute distress.

**Figure 1:**
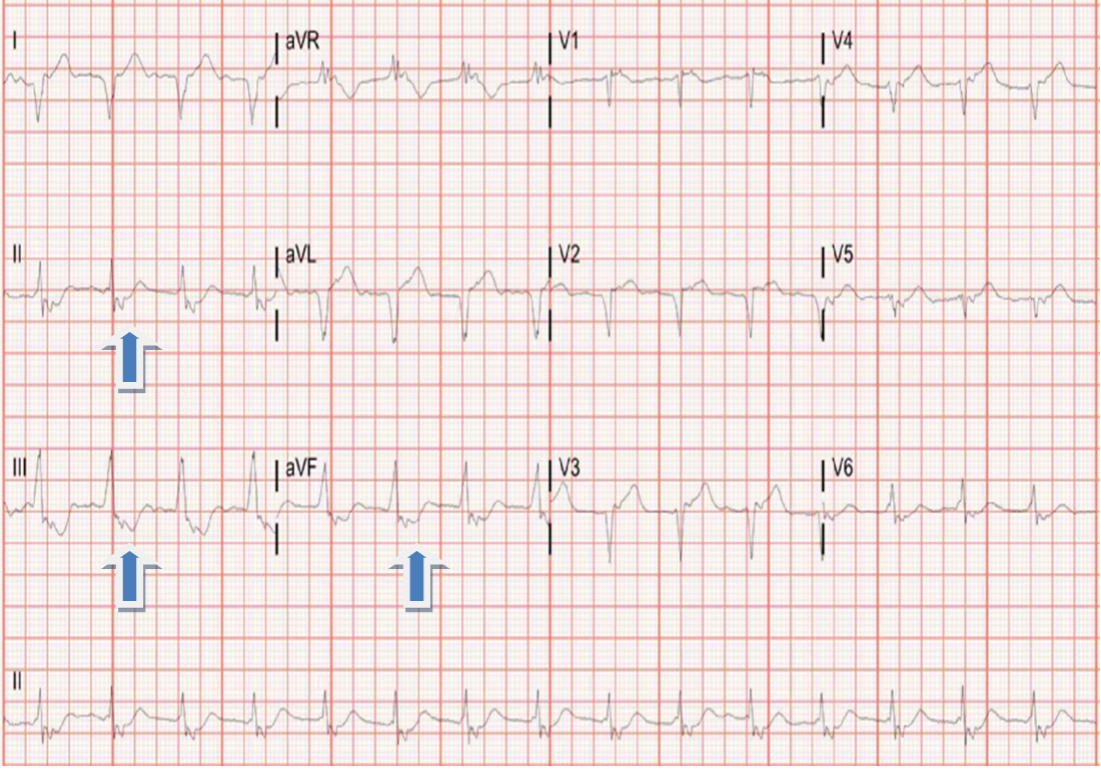
EKG showing ST depression in the inferior leads (II, III, AVF) with possible prior anterior-septal infarction.

Diagnostic testing in the emergency department showed abnormal findings of elevated troponin level of 12.5 ng/ml, which at the facility considered normal levels to be less than 0.039 ng/ml. Troponins are enzymes that are measured in the blood stream and when are present, it suggests that there is heart damage. CT pulmonary angiogram was obtained and the results were unremarkable for pulmonary embolism, a condition where there is blood clots in the lungs. Initial EKG obtained on presentation showed ST depression in the inferior leads with possible prior anterior-septal infarction. (Figure 1) The inferior leads are II, III and AVF on an EKG as delineated by the blue arrows on the figure. Although this does not mean there is a heart attack occurring at this time, it does mean that there is ischemia to the heart muscle.

**Figure 2:**
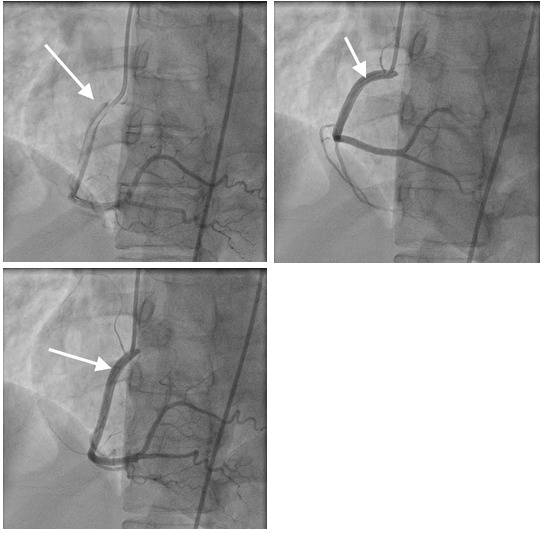
Dissection of the right coronary artery is shown in all views of angiogram.

The patient was taken to the catheterization lab by the cardiology team. The result of the procedure was consistent with spontaneous dissection of the right coronary artery. (Figure 2) There was no intervention performed. The cardiothoracic surgeon who decided that there is no need for surgical intervention also evaluated the patient. She was medically managed and discharged home with aspirin, atorvastatin, metoprolol, and Plavix. Her hospital course was uncomplicated and she was discharged home in stable condition.

## DISCUSSION

Spontaneous coronary artery dissection is a phenomenon becoming more recognized in the recent literature as a cause of ACS. The typical patient to develop this condition is more commonly to have a history such as the one presented in this case report. In one study that contained a large series patient sample over three decades with spontaneous coronary artery dissection, the mean age was 43 years and 82 percent were women.^4^ Postpartum status was present in 18 percent of the women.

It has been well known that during pregnancy many hormonal changes occur including several cardiocirculatory effects. There is loss of normal elastic fibers and increase in fragmentation of reticular fibers that reduce wall strength which may lead to the dissection.^5^ There is also an increase in the risks of developing arterial dissection in women with chronic hormonal pregnancy and multiple births.^6^ Although there is no single disease that is known to cause spontaneous coronary artery dissection, there are additional associated factors such as systemic inflammatory and vascular disorders.^7^

Fibromuscular dysplasia is well known to be associated with spontaneous coronary artery dissection.^8^ Other possible factors associated with this rare phenomenon include migraines and tortuosity of the coronary arteries. Tortuosity is the bending and twisting of vessels that commonly occurs in the human body. In one retrospective study with 40 Australian patients with spontaneous coronary artery dissection, migraines were reported in 43 percent of the patients.^8^ Another retrospective study showed tortuosity to be more often present in those with dissections.^9^ However, this cannot be determined as a true cause due its presence in other vasculopathies such as fibromuscular dysplasia. Due to these common associated diseases, patients with spontaneous coronary artery dissection should be screened for fibromuscular dysplasia and involvement of other vessels such as the iliac, renal and cerebral arteries.

The patient in this case report had dissection of the right coronary artery, which correlated with the EKG changes seen. However, this is not the artery most commonly involved in spontaneous coronary artery dissection. The left anterior descending is the most frequently affected vessel in this disease.^10^ The right coronary artery is also very common but more so in men.^11^ Spontaneous coronary artery dissection may also present in men in any form of ACS. A case series study reported five patients in south Asia 1994 to 2015 in which four patients were young men.^12^ They were managed conservatively with medications only and no surgical intervention was performed. Each of these patients had a favorable long-term prognosis.

There are no current guidelines for the management of spontaneous coronary artery dissection due to limited data and clinical experience. Similar to acute myocardial infarction, various treatments include conservative management, percutaneous coronary artery intervention (PCI), and coronary artery bypass grafting and fibrinolysis may be used. Most cases with spontaneous coronary artery dissection are preferred to be managed conservatively.^13^ This includes use of beta blockers, aspirin, Plavix and statins as were used in the patient’s case. In patients with ongoing ischemia or those that are hemodynamically unstable, the use of PCI or surgery may be required. There is high complication rate with PCI despite similar five-year outcomes when compared with conservative management. The PCI failure rate have shown to be as high as 53% in some studies.^13^ Fibrin thrombolytic therapy is not recommended either due to it resulting in progression of dissection.^14^

The overall prognosis in patients with spontaneous coronary artery dissection varies and there is a high rate of recurrent events. Multiple retrospective studies have been used to demonstrate the rate of recurrence with wide range of results. Another study of 75 patients report a reoccurrence rate 24 percent over a 15-year period.^15^ However, another study showed an estimated ten year rate of death and dissection recurrence was as high as 47 percent.^4^ It is clear that patients with spontaneous coronary artery dissection must have long term follow up and should be educated on their condition. Although these patients can present with just chest pain alone. They may also have a heart attack presentation with multiple symptoms such as nausea, vomiting, difficulty in breathing, dizziness or even present in arrest.

## CONCLUSIONS

Spontaneous coronary artery dissection is somewhat rarer phenomenon that should be considered as a possible cause of ACS. Pregnant women or those in the postpartum period are at higher risk to develop this condition. Therefore, the patient presented in this case report can be considered a classic case. Although there is literature concerning the cause and management of this disease, there are still many more questions that need to be answered regarding this phenomenon. Guidelines concerning standard management have yet to be determined. To date, medical management has been viewed as a standard of care approach unless a patient is hemodynamically unstable or their symptoms persist. Long-term follow up remains key to optimal patient care and patients should be educated concerning their prognosis and potential recurrence risks.

### Conflict of Interest

The authors declare no conflict of interest.
